# Supplementary material for: Social Media Polarization and Echo Chambers in the Context of COVID-19: Case Study
Source: JMIRx Med. 2021 Aug 5;2(3):e29570. doi: 10.2196/29570 (PMC8371575; doi:10.2196/29570)
Supplement: Multimedia Appendix 1 [file xmed_v2i3e29570_app1.pdf]

Social Media Polarization and Echo Chambers in the Context of COVID-19: Case Study Supplementary Material

Julie Jiang, Xiang Ren, and Emilio Ferrara

Model Type	Model	Profile	Network	Acc.	AUC
Average word embeddings	GloVe-wiki-gigaword-300	✓	✗	0.856	0.875
	Word2Vec-google-news-300	✓	✗	0.852	**0.877**
Average transformer output	BERT-base-uncased	✓	✗	0.859	0.882
	BERT-large-uncased	✓	✗	0.862	0.885
	DistilBERT-uncased	✓	✗	0.863	0.888
	RoBERTa-base	✓	✗	0.870	0.898
	RoBERTa-large	✓	✗	0.882	**0.914**
Fine-tuned transformers	BERT-base-uncased	✓	✗	0.900	**0.932**
	DistilBERT-uncased	✓	✗	0.899	0.931
	RoBERTa-base	✓	✗	0.893	0.916
S-BERT	S-BERT-large-uncased	✓	✗	0.869	0.890
	S-DistilBERT-uncased	✓	✗	0.864	0.885
	S-RoBERTa-large	✓	✗	0.879	**0.903**
Network embedding	node2vec*	✗	✓	0.928	**0.955**
	GraphSAGE + RoBERTa-base	✓	✓	0.789	0.725
Retweet-BERT (our model)	Retweet-DistilBERT-one-neg	✓	✓	0.900	0.933
	Retweet-DistilBERT-mult-neg	✓	✓	0.935	0.965
	Retweet-BERT-base-mult-neg	✓	✓	0.934	<u>**0.966**</u>

Table 1: 5-fold CV results for political leaning classification on seed users for various models that are tuned via grid-search ($N = 79$k). We indicate whether each model makes use of the profile descriptions and retweet network structure. The best AUC score for each model type is shown in bold and the best overall scores are underlined. *node2vec, an inductive-only model, can only be applied to non-isolated users in the retweet network.
